# Early radiological outcomes of a fully porous bridging collar in lower-limb endoprosthetic reconstructions: a case-matched retrospective series to assess osseointegration

**DOI:** 10.1186/s42836-023-00230-2

**Published:** 2024-03-02

**Authors:** Jonathan Stevenson, M. Ather Siddiqi, Vicky Sheehy, Ben Kendrick, Duncan Whitwell, Adrian Taylor, Gordon Blunn, Hasan R. Mohammad, Atul F. Kamath, Sofia Thoma

**Affiliations:** 1 Royal Orthopaedic Hospital NHS Foundation Trust, Bristol Road South, Northfield, Birmingham, B31 2AP UK; 2 Aston University Medical School, Aston University, Birmingham, B4 7ET UK; 3 Nuffield Orthopaedic Centre, Oxford University Hospitals Trust, Windmill Road, Oxford, OX3 7LD UK; 4 School of Pharmacy and Biomedical Sciences, University of Portsmouth, St. Michaels Building, White Swan Road, Portsmouth, PO1 2DT UK; 5 Bart’s Bone Joint Health, Bart’s and the London School of Medicine and Dentistry, Blizard Institute, 4 Newark St., London, E1 2AT UK; 6 Cleveland Clinic Foundation, Orthopaedic and Rheumatologic Institute, 9500 Euclid Avenue A40, Cleveland, OH 44195 USA

**Keywords:** Endoprosthetic replacement, Porous collar, Bone ongrowth, Aseptic loosening

## Abstract

**Background:**

Limb-salvage surgery involving the utilization of endoprosthetic replacements is commonly employed following segmental bone resection for primary and secondary bone tumors. This study aimed to evaluate whether a fully porous bridging collar promotes early osseous integration in endoprosthetic replacements.

**Methods:**

We undertook a retrospective review of all lower-limb endoprostheses utilizing a fully porous endosteal bridging collar design. We matched this cohort with a conventional extra-osteal non-porous fully hydroxyapatite-coated grooved collar cohort according to surgical indication, implant type, resection length, age, and follow-up time. At 6, 12, and 24 months post-implantation, radiographs were assessed for the number of cortices with or without osseointegration on orthogonal radiographs. Each radiograph was scored on a scale of -4 to + 4 for the number of cortices bridging the ongrowth between the bone and the collar of the prosthesis. Implant survival was estimated using the Kaplan–Meier method, and the mean number of osseointegrated cortices at each time point between the collar designs was compared using a paired *t*-test.

**Results:**

Ninety patients were retrospectively identified and analyzed. After exclusion, 40 patients with porous bridging collars matched with 40 patients with conventional extra-osteal non-porous collars were included in the study (*n* = 80). The mean age was 63.4 years (range 16–91 years); there were 37 males and 43 females. The groups showed no difference in implant survival (*P* = 0.54). The mean number of cortices with radiographic ongrowth for the porous bridging collar and non-porous collar groups was 2.1 and 0.3, respectively, at 6-month (*P* < 0.0001), 2.4 and 0.5, respectively, at 12-month (*P* = 0.044), and 3.2 and -0.2, respectively, at 24-month (*P* = 0.18) radiological follow-up.

**Conclusion:**

These findings indicate that fully porous bridging collars increased the number of cortices, with evidence of bone ongrowth between 6 and 24 months post-implantation. By contrast, extra-osteal collars exhibited reduced evidence of ongrowth between 6 and 24 months post-implantation. In the medium term, the use of a fully porous bridging collar may translate to a reduced incidence of aseptic loosening.

**Graphical Abstract:**

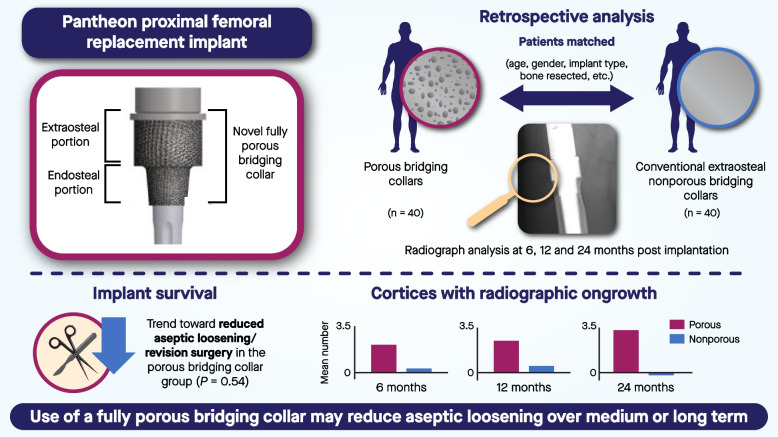

## Introduction

Limb salvage surgery using endoprosthetic replacements (EPRs) is frequently used after segmental bone resection for primary and secondary bone tumors. EPRs are also increasingly considered for failed osteosynthesis and revision arthroplasties with significant bone loss [1–4]. EPRs, as opposed to biological reconstructions, allow for early weight bearing, shorter operative times, and decreased risk of infection and disease transmission. These factors make them a particularly good option for elderly-stage patients and those with medical co-morbidities [5].

Due to improved survival in patients with primary bone sarcoma and metastatic bone disease due to advances in chemotherapy, radiotherapy, and immunotherapy, modern EPRs require greater implant survival to minimize the risk of revision surgery. A particular concern is long-term EPR survival in young patients, whose greater physical demands place higher stress on the implant cement and cement bone interfaces [1, 6]. Additional considerations for implant survival of EPRs include, but are not limited to, the length of bone resected [7], soft-tissue available to support the reconstructions, and concomitant morbidity associated with chemotherapy, irradiation, and prosthetic joint infection.

Common causes of EPR failure necessitating revision were classified by Henderson et al. as mechanical, including soft-tissue attachment failure; aseptic loosening; structural implant failure; and non-mechanical failure, including local recurrence and prosthetic joint infection (PJI) [1, 6, 8]. Some of the benefits of EPRs include their immediate availability, achievement of instant fixation, and the advantage of early weight-bearing and mobilization. The leading cause of EPR failure is aseptic loosening. In a study of 661 EPRs, aseptic loosening accounted for 25% of revisions [1]. Two other studies reported similar aseptic loosening to be the major failure mechanism of EPRs, with rates of loosening of 2.9% and 28.6% after 4 and 10 years, respectively [9, 10].

Aseptic loosening of EPRs is associated with the loss of cortical bone (osteolysis). The process initiates at the bone-prosthesis interface and progresses along the stem [10]. This could be countered, in theory, by having a region of bone ongrowth over the shoulder of the prosthesis at the bone-prosthesis interface to promote osseointegration, a process described as extracortical bone bridging [11, 12]. This process has the potential to reduce the risk of aseptic loosening by improving stress transfer between the implant and bone, as well as providing an effective “seal”, preventing entry of joint fluid and debris into the cement–bone interface, thereby preventing aseptic loosening.

Historically, hydroxyapatite (HA)-coated collars have been shown to enhance osseointegration. Histological analysis demonstrated lamellar bone was in direct contact with HA-coated grooves, and bony bridging occurred in some cases [12]. A patient-matched study confirmed that the osseointegration of HA collars reduced the development of radiolucent lines around a cemented stem [13]. Histological analysis of non-HA porous coated implants identified fibrous bridging tissue rather than osseointegration, despite radiographs indicating ongrowth at the bone-implant interface when using porous coated implants [14].

It is thought that a more porous structure may permit greater osseointegration with the host bone [15]. Although block porous metal has been used successfully to treat metaphyseal defects around revision arthroplasties of the knee [16], to our knowledge, there are no reports on using this to encourage osseointegration in EPRs. Animal models have demonstrated that a 3-D printed titanium porous collar allowed for osseointegration and was superior to current grooved HA-coated designs, which rely on surface ongrowth for osseointegration [11]. Importantly, the 3-D printing technique allows for fully porous implants and does not rely on porous coatings.

In this study, we investigated the use of a novel fully porous collar design with lower extremity EPR reconstructions to achieve osseointegration. We presented a comparison of this technology to determine radiographic osseointegration and implant survival between conventional non-porous collared EPRs and novel fully porous endosteal collared EPRs.

## Materials and methods

This study included 90 patients operated on for segmental bone loss following resection of bone sarcomas, metastatic bone disease, or revision arthroplasty of the lower limb. In every case, reconstruction was done with a cemented modular implant system utilizing a porous collar (Pantheon, AdlerOrtho S.p.A, Milan, Italy). All procedures were performed by the same group of surgeons at each centre.

**Fig. 1:**
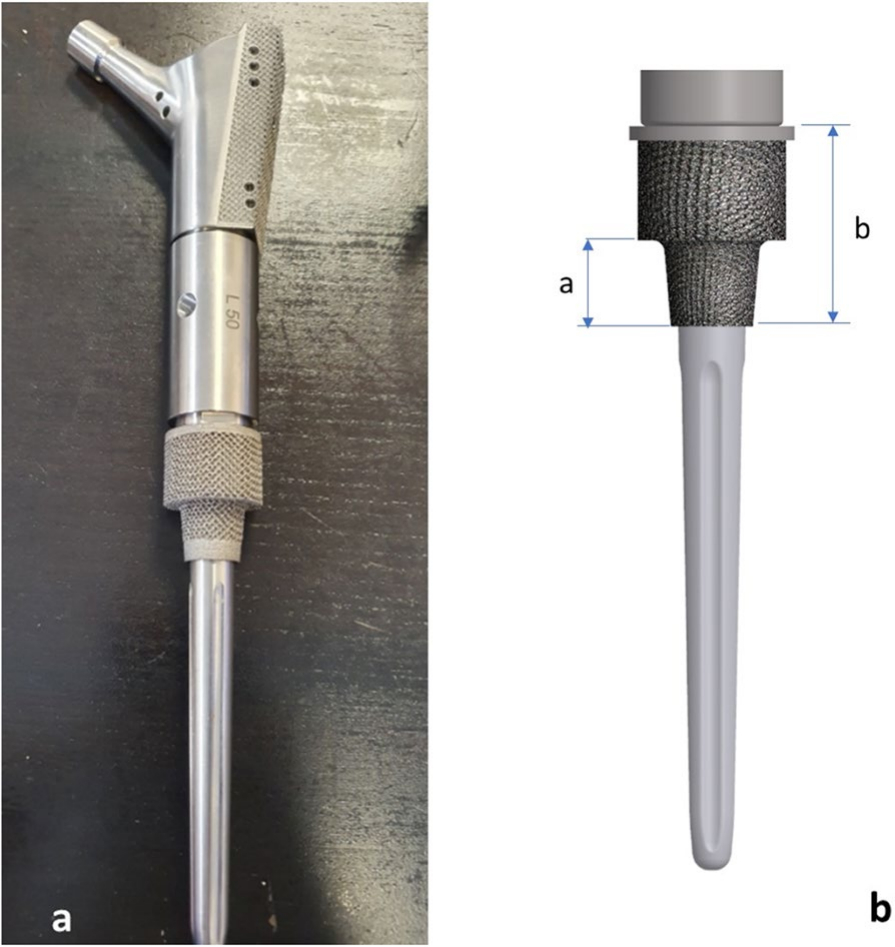
**a** The Pantheon PFR implant; **b** Diagrammatic representation showing (**a**) the endosteal portion of the fully porous collar, and (**b**) the extra-osteal and endosteal porous bridging collar

The study group was case-matched for age, gender, implant type, comorbid conditions, indication for surgery (Table 1), institution, and length of bone resected from a cohort of patients from the existing institutional databases who had been treated with another cemented modular implant system utilizing a non-porous collar (METS, Stryker, Elstree, UK). This non-porous system has a well-established history in reconstructions of lower-limb bone defects and consists of an extra-osteal HA-coated non-porous collar. The fully porous collared EPR system has been used for five years at the authors’ institutions and consists of a fully porous 3D-printed titanium bridging collar, which also has an endosteal porous section that achieves immediate press fit into the meta-diaphyseal bone (Fig. 1). This collar is manufactured from titanium alloy (Ti6Al4V) with a pore size of 1,000 microns manufactured by 3D printing using additive manufacturing.

**Table 1:** Indications for surgery in both groups

Indication	Porous bridging	Non-porous	Total
Infection	15	13	28
Aseptic loosening	5	6	11
Periprosthetic fracture	4	5	9
Primary bone tumour	8	8	16
Metastatic bone disease	8	8	16
Total	40	40	80

All patients eligible for inclusion in this study underwent surgical procedures to address segmental bone loss resulting from the resection of bone sarcomas, metastatic bone disease, or revision arthroplasty in the lower limbs. These surgeries were exclusively performed at two renowned referral hospitals in the UK: the Nuffield Orthopaedic Centre in Oxford and the Royal Orthopaedic Centre in Birmingham. We enrolled both male and female individuals aged 16 years or older who were willing and able to provide informed consent for the surgical interventions.

As part of our exclusion criteria, we excluded participants who were either unwilling or unable to provide informed consent for the surgical procedures. We also excluded individuals who were missing 6-month follow-up radiographs from our analysis.

### Radiographic analysis

**Fig. 2:**
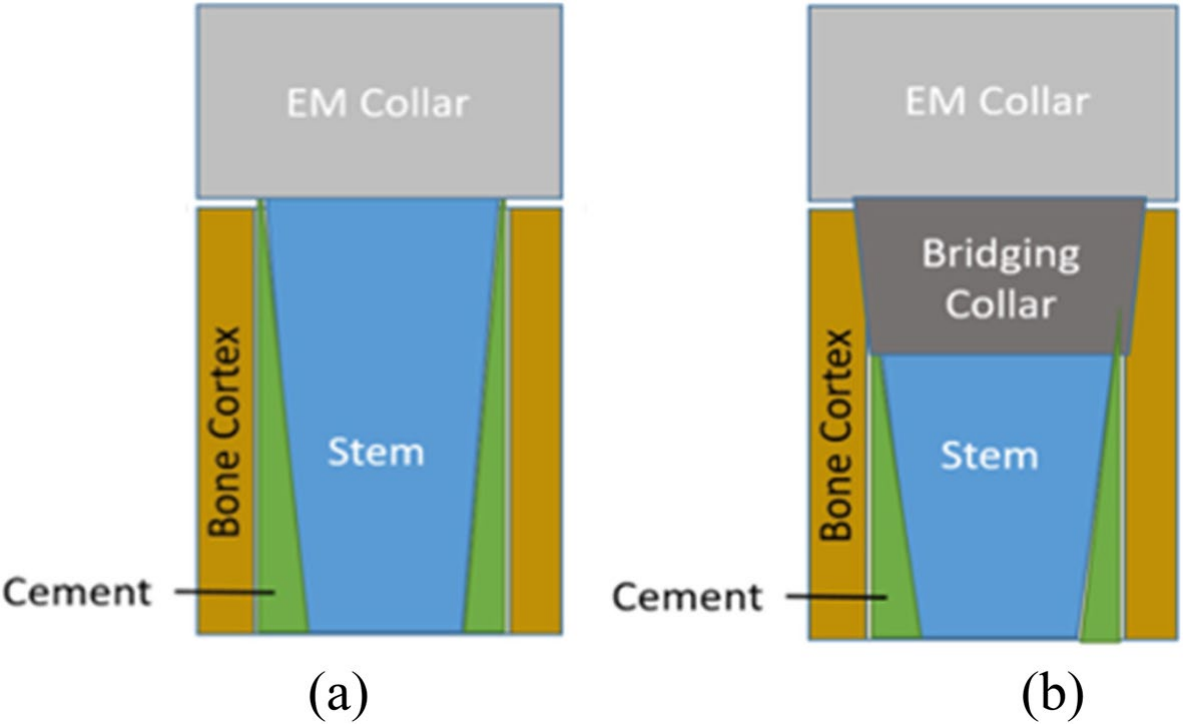
Diagrammatic representation of the radiographic ongrowth score for endoprostheses (ROSE) for the (**a**) non-porous collar and the (**b**) porous bridging collar

Radiographs at 3, 6, 12, and 24 months post-implantation follow-ups were assessed by two independent assessors for the number of cortices on orthogonal radiographs showing bone ongrowth and/or osteolysis. The orthogonal radiographs were direct lateral and anterior–posterior and were taken according to standard radiographic procedures for each centre. The radiographical analysis was based on a modification of a previously published method [12] (Fig. 2). The radiographs were viewed and assessed at each centre from the respective hospital PACS system.

Extracortical bone bridging osseointegration onto the endoprosthetic collars was quantified radiographically using the radiographic ongrowth score for endoprostheses (ROSE). Osseointegration was quantified in four zones: the medial and lateral aspects on the anteroposterior radiographs and the anterior and posterior aspects on the lateral radiographs. The bone that was separated from the collar at the resection site by a clear radiolucent line was not considered on-grown and was assigned a score of 0. This score was also assigned to all implants on immediate post-implantation radiographs.

**Fig. 3:**
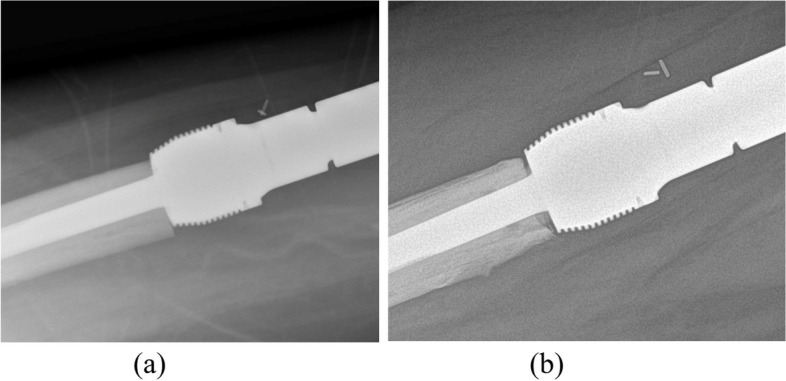
Example of radiographic ongrowth score for endoprostheses (ROSE) using the extra-osteal non-porous collar: **a** radiograph immediately post-implantation showing the osteotomized bone abutting the collar without radiolucencies, and **b** radiograph after 24 months demonstrating radiolucencies anteriorly and posteriorly between the host bone and non-porous collar, indicating a ROSE score of -2 on this radiograph

**Fig. 4:**
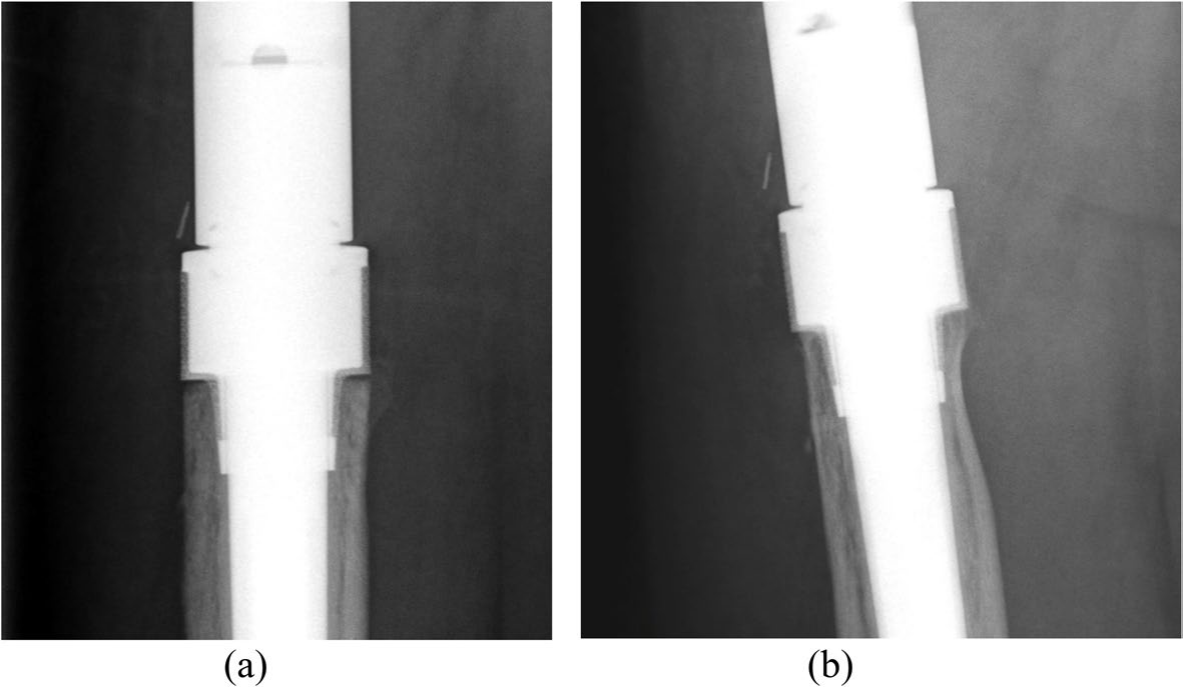
Example of radiographic ongrowth score for endoprostheses (ROSE) using the porous bridging collar: **a** radiograph immediately post-implantation showing radiolucencies between the osteotomized bone and the extra-osteal portion of the porous collar; **b** absence of radiolucencies between the host bone and the collar medially and laterally, indicating a ROSE score of + 2 on this radiograph

For follow-up imaging, a radiographic score of 1 represented bone in contact with the collar at the resection site in any of the four (anterior, posterior, medial, and lateral) zones. A score of -1 represented no bone in contact with the collar at the resection site and bone *resorption* in any of the four zones (Fig. 3). The maximal score was 4, which represented bone in contact with the collar at the resection site in all four zones (Fig. 4). The minimal score was -4, representing no bone ongrowth and no bone contact with the collar at the resection site, or resorption in any of the four zones.

### Surgical technique

After adequate resection of the bone and reaming of the canal, endosteal reaming was performed to allow the collar to sit tightly in the bone. For the porous collared prosthesis, face reamers were used to facilitate flush fitting of the prosthesis on the cut surface. A trial reduction was performed to confirm the optimal implants required. For cemented implantation, the canal was washed and brushed, and a cement restrictor was applied at the desired level. The canal was then retrogradely washed and dried with ribbon gauze. Cement was inserted retrogradely using a cement gun with a third-generation technique. In the porous collared system, it was important to pay close attention to the cementing technique to avoid cement penetration into the porous regions, promoting bone contact and to achieve good osteointegration in the porous collar. To avoid cement being introduced into the endosteal section of the porous collar, cement was only introduced into the canal within approximately 3 cm of the cut surface to allow it to rise under pressurisation to the bottom of the collar. A specific displacement instrument was used to allow accurate filling of the intramedullary canal prior to inserting the stem and endosteal collar to ensure that no cement was interposed in the collar-bone interface. The bridging non-porous collar has a non-porous end to divert the cement away from the porous surface.

All patients underwent a standard physiotherapy protocol that involved either partial or full weight bearing for six weeks (at the discretion of the surgeon) postoperatively, followed by full weight bearing thereafter. All patients received thromboprophylaxis for either two or four weeks postoperatively in the form of subcutaneous low molecular weight heparin injections, unless restarted on warfarin or other new oral anticoagulants.

### Statistical analysis

Case matching was based on propensity score matching. The first step involved estimating propensity scores for everyone in the dataset. Propensity scores were obtained using a logistic regression model, in which the binary treatment assignment was regressed on the covariates of sex, age, resection length, and indication for surgery. These covariates were selected based on their relevance and potential influence on both the treatment assignment and the outcome. Once the propensity scores were computed, a nearest neighbour matching algorithm was used to pair individuals from the treatment group with their closest counterparts in the control group based on similar propensity scores. A standardized mean difference (SMD) of 0.1 or less was considered to indicate a good balance between the treatment and control groups for a particular covariate, whereas an SMD > 0.1 was considered to indicate an unbalanced distribution [17]. Following matching, implant survival was estimated using the Kaplan–Meier method, and the two different collar design cohorts were compared using the log-rank test. A mean ROSE score was calculated for each group at each time point and presented as a box and whisker plot. A paired* t*-test was used to compare ROSE scores between the two groups at each postoperative timepoint [18]. All statistical calculations were performed using SPSS version 20.0.

## Results

Ninety patients were retrospectively identified and analyzed. After case matching and exclusion, 40 porous cases with a mean follow-up of 1.7 years (range 0.6–4.8 years) were compared with a matched cohort of 40 non-porous patients with a mean follow-up of 2.0 years (range 0.5–5.6 years) (*P* = 0.16). In the fully porous group, there were 23 proximal femoral replacements (PFRs), 15 distal femoral replacements (DFRs), and two proximal tibial replacements (PTRs). In the non-porous group, there were 24 PFRs, 15 DFRs, and 1 PTR.

The common indications for surgery were revision of a failed hip or knee arthroplasty for PJI (*n* = 28), *en bloc* resection and reconstruction of a primary bone tumour (*n* = 16), resection and reconstruction for metastatic bone disease (*n* = 16), revision of a failed hip or knee arthroplasty due to aseptic loosening (*n* = 11), and peri-prosthetic fracture (*n* = 9) (Table 1).

**Fig. 5:**
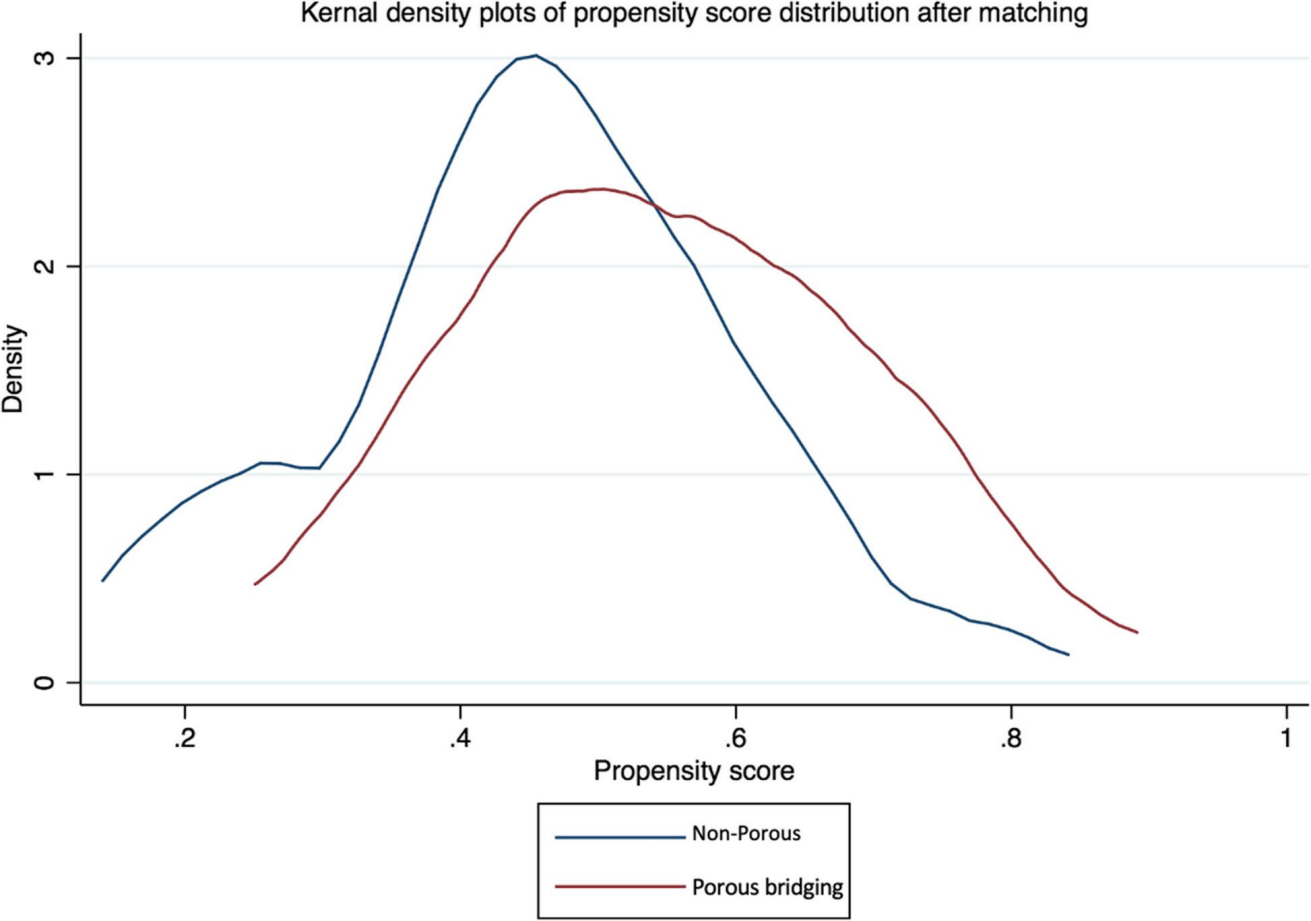
Propensity score distribution of the comparative groups

Mean age was 63.4 years (range 16–91 years). There were 37 males and 43 females. There were 46 proximal femoral, 30 distal femoral, and four proximal tibial endoprosthetic replacements. The baseline characteristics of the analyzed cohort were assessed using logistical regression with covariates, including sex, age, resection length, and indication for surgery. A propensity score distribution was generated, showing the similarities between the groups, which were considered reasonably well-matched (Fig. 5). The groups were reasonably well matched for baseline characteristics, as two of those, resection length and age, showed a relevant difference, and one (indication) showed a minor imbalance (Table 2).

**Table 2:** Propensity-matched data

Variable	Implant type		SMD
	Non-porous (*n* = 40)	Porous Bridging (*n* = 40)	
Indication			0.132
Aseptic loosening	6 (15.0)	5 (12.5)	
Metastatic bone disease	8 (20.0)	8 (20.0)	
Primary bone tumour	8 (20.0)	8 (20.0)	
Periprosthetic joint infection	13 (32.5)	15 (37.5)	
Periprosthetic fracture	5 (12.5)	19 (47.5)	
Male sex	18 (45.0)	19 (47.5)	0.05
Resection length [mm]^a^	155.6 ± 61.6	178.3 ± 61.1	0.367
Age [years]^a^	66.6 ± 19.6	60.3 ± 19.2	0.327

Frequencies expressed as number of observations (percentage) for all outcomes (^a^except resection length/age which are expressed as mean ± standard deviation (SD))

*SMD* standardized mean difference

**Fig. 6:**
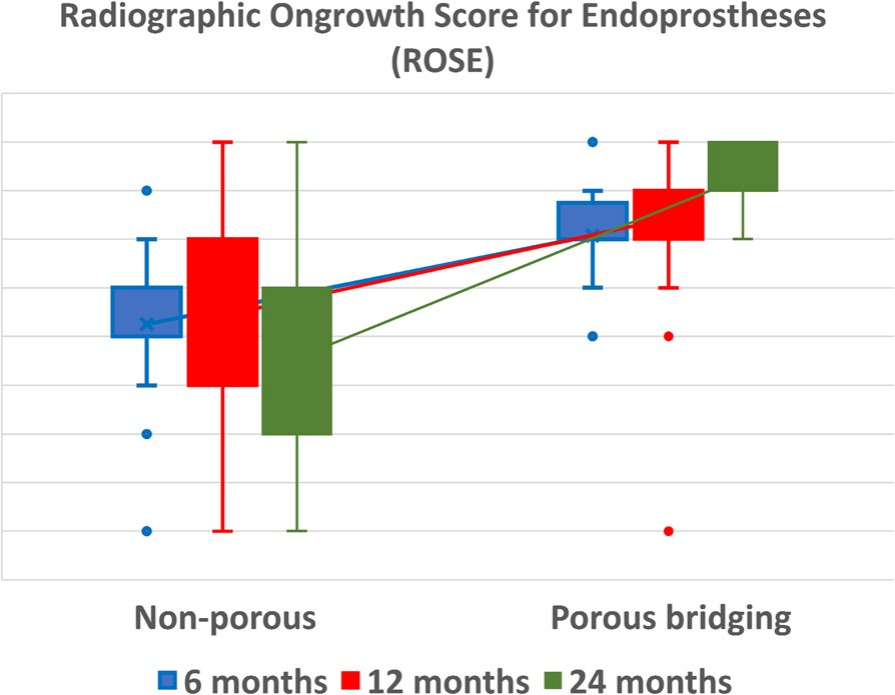
Radiographic ongrowth score for endoprostheses (ROSE) for the non-porous collar (left) and porous bridging collar (right) groups at 6, 12, and 24 months. Boxes represent interquartile ranges, whiskers represent non-outlier ranges, and dots represent outliers

After six months of radiological follow-up, the mean number of cortices (± SD) with radiographic ongrowth (ROSE score) for the porous bridging collar (*n* = 37) and non-porous collar groups (*n* = 36) was 2.1 ± 1.1 and 0.3 ± 1.1, respectively (*P* ≤ 0.0001). After 12 months, the mean ± SD ROSE scores for the porous bridging collar (*n* = 18) and non-porous collar groups (*n* = 31) were 2.4 ± 1.9 and 0.5 ± 1.5, respectively (*P* = 0.044). After 24 months, the mean ROSE ± SD scores for the porous bridging collar (*n* = 5) and non-porous collar groups (*n* = 20) were 3.2 ± 0.9 and -0.2 ± 2.0, respectively (*P* = 0.18) (Fig. 6).

**Fig. 7:**
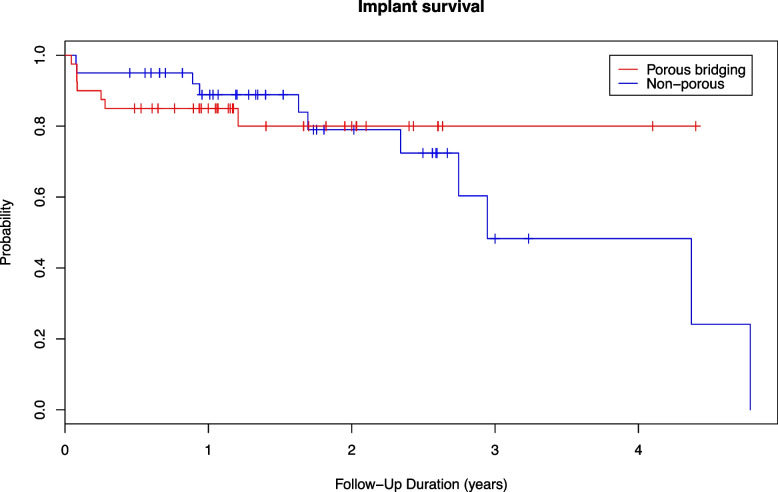
Kaplan–Meier analysis of implant survival showing no statistically significant difference (*P* = 0.54) between the non-porous collar (left) and porous bridging collar (right) groups in estimated implant survival, with revision surgery as an end point

Implant survival showed no difference between the groups (*P* = 0.54) (Fig. 7). Of the 40 porous bridging collar EPRs, six were revised for recurrent PJI and one for instability of the hip (total seven), whereas four of the non-porous EPRs were revised due to recurrent PJI three (3) for aseptic loosening, and one (1) for periprosthetic fracture (PPF) (total eight). There were no cases of aseptic loosening in the porous collar group.

## Discussion

Long-term survival of EPRs after failed arthroplasty or oncology surgery continues to be a challenge. Although these implants are widely used, the rate of revision for any reason remains five to ten times higher than the rates seen following routine total joint arthroplasties [13, 19]. Osseointegration of the collar of EPRs has been associated with a more advantageous biomechanical environment and improved stem fixation, especially when HA-coated collars are used [2, 3]. When bone is osseointegrated with the collar of the prosthesis, survival at 10 years is increased by more than 20% [12]. However, obtaining adequate osseointegration at the collar-bone interface is often difficult, and rates of aseptic loosening remain high.

Osseointegration is known to reduce aseptic loosening, possibly by sealing the bone-prosthesis interface from wear debris and synovial fluid [11, 20]. The novel fully porous collar design is thought to osseointegrate through the direct ingrowth of bone into the pores of the porous titanium alloy collar. In this hybrid system, an endosteal porous sleeve engages the endosteum to promote endosteal osseointegration, rather than relying solely on periosteal integration. The sleeve also increases the effective bone-collar contact surface area and simultaneously provides a tight press fit for early stability and weight bearing. Cementing part of the stem also ensures stability for better osseointegration. Although many studies have examined bone ingrowth on porous implants [16], the present study is the first to compare in vivo retrospective data of this fully porous bridging collar to a conventional non-porous HA collar in endoprosthetic replacement surgery.

There were failures in both patient groups. However, it is interesting to note that the only failures in the porous collar group were for PJI after the second-stage re-revision of chronic infections, which is known to carry a high relapse rate [21]. Whereas the non-porous group had two revisions due to aseptic loosening, the porous collar group had none. Overall, there was no statistically significant implant survival difference between groups after short-term follow-up (Fig. 5), although the survival curves suggest a trend towards reduced aseptic loosening in the porous group.

At the 6- and 12-month radiographic follow-up, however, there was significantly more radiographic ongrowth (indicating osseointegration) in the porous collar group than in the non-porous group, suggesting that this novel porous design is superior in achieving the osseointegration associated with reduced risk of aseptic loosening, which is surprising after only short follow-up. This finding was not evident at the 24-month follow-up, which is attributed to the smaller number of cases with 24-month follow-up radiographs. Thus, further investigation of medium-term outcomes is needed. We posit that the endosteal portion of the porous collar is likely to have a significant effect on osseointegration, as bone formation in this region may reduce stress on the periosteal surface, reducing extracortical bone formation. This has also been demonstrated theoretically, with researchers finding that, for proximal femoral replacements, the use of a porous endosteal collar resulted in preferential bone loading and a lower likelihood of bone resorption when compared to a collar without the endosteal portion [20]. Definite confirmation of this phenomenon can only be proven by a large retrieval study, and it is still too early in the development of this collar to have such data available. However, the short-term radiographic and survivorship outcomes support the continued investigation of this novel porous collar design with its endosteal sleeve.

There are several limitations to the study, including the small cohorts, short-term follow-up, and confounding results from the observational character of the study. The retrospective and case-matched rather than randomised nature of the study inevitably led to a higher probability of introducing bias that may have jeopardized the validity of our findings. Care was taken to carefully match the groups to minimize potential biases from heterogeneous indications and patient demographics, which are common in endoprosthetic replacement studies. However, the groups were not perfectly balanced due to the small source population from which the study patients were sampled. For this study, the differences in resection length, although not exactly matched, were very similar, and the differences in age were as expected for this type of matched cohort, which is a strength of the study, lending credibility to the validity of the findings. We do not feel that the minimal differences in age and resection length would have had any bearing on the study outcomes, particularly early radiographic osseointegration.

However, it is difficult to determine the exact relevant differences between the two prostheses with this type of study and its limitations. Importantly, the cementing technique was the same for both groups, with a limited amount of cement being used in the porous group to avoid cement interfering with endosteal sleeve osseointegration. The overall design parameters are similar between all cemented EPR stems, with large polished alloy cylinders being used for the reconstruction length. The only major difference in design is at the bone-implant junction, having the most significant difference between the two prostheses studied: a fully porous collar with an endosteal sleeve and a conventional non-porous HA-coated collar. We suppose that this might explain the early difference shown in early osseointegration; however, further study is required to investigate this conclusively.

Although osseointegration has been used as the radiological outcome in this study and previous ones, the precise quantity of bone growth in a porous structure cannot be measured solely by measuring cortices. This measurement requires a retrieval study or, at best, a quantitative computed tomography analysis (QCT). This was beyond the scope of the current study. A further study is currently randomizing patients to establish radiological outcomes and implant survivorship in the medium term.

## Conclusion

Our findings showed that fully porous bridging collars increased the number of cortices, with evidence of bone ongrowth between 6 and 24 months of follow-up. By contrast, extra-osteal collars exhibited reduced evidence of ongrowth between 6 and 24 months of follow-up. In the medium term, the use of a fully porous bridging collar may translate into a reduced incidence of aseptic loosening.

## Data Availability

The datasets used and/or analyzed during the current study are available from the corresponding author upon reasonable request.
